# Sorption Characteristics and Chromatographic Separation of ^90^Y^3+^ from ^90^Sr^2+^ from Aqueous Media by Chelex-100 (Anion Ion Exchange) Packed Column

**DOI:** 10.1155/2024/6232381

**Published:** 2024-05-13

**Authors:** Mohammad S. El-Shahawi, Hassan Alwael, Abdulaziz A. Alsibaai, Abdelgany Hamza, Faisal K. Algethami, Fatmah M. Alshareef, Sanaa H. El-Khouly, Neven Eweda

**Affiliations:** ^1^Department of Chemistry, Faculty of Science, King Abdulaziz University, P.O. Box 80203, Jeddah 21589, Saudi Arabia; ^2^Department of Chemistry, College of Science, Imam Mohammad Ibn Saud Islamic University (IMSIU), P.O. Box 90950, Riyadh 11623, Saudi Arabia; ^3^Department of Production of Isotopes and Generators, Atomic Energy Authority, Cairo, Egypt

## Abstract

There is growing demand for separation of ^90^Y carrier free from ^90^Sr coexisting to produce high purity ^90^Y essential for radiopharmaceutical uses. Thus, in this context the sorption profiles of Y^3+^ and Sr^2+^ from aqueous solutions containing diethylenetriaminepenta acetic acid (DTPA), ethylenediaminetetra-acetic acid (EDTA), acetic acid, citric acid, or NaCl onto Chelex-100 (anion ion exchange) solid sorbent were critically studied for developing an efficient and low-cost methodology for selective separation of Y^3+^ from Sr^2+^ ions (1.0 × 10^−5^ M). Batch experiments displayed relative chemical extraction percentage (98 ± 5.4%) of Y^3+^ from aqueous acetic acid solution onto Chelex-100 (anion ion exchanger), whereas Sr^2+^ species showed no sorption. Hence, a selective separation of Y^3+^ from its parent ^90^Sr^2+^ has been established based upon percolation of the aqueous solution of Y^3+^ and Sr^2+^ ions containing acetic acid at pH 1-2 through Chelex-100 sorbent packed column at a 2 mL min^−1^ flow rate. Y^3+^ species were retained quantitatively while Sr^2+^ ions were not sorbed and passed through the sorbent packed column without extraction. The sorbed Y^3+^ species were then recovered from the sorbent packed column with HNO_3_ (1.0 M) at a 1.0 mL min^−1^ flow rate. A dual extraction mechanism comprising absorption associated to “weak-base anion exchanger” and “solvent extraction” of Y^3+^ as (YCl_6_)^3−^ and an extra part for “surface adsorption” of Y^3+^ by the sorbent is proposed. The established method was validated by measuring the radiochemical (99.2 ± 2 1%), radionuclide purity and retardation factor (*R*_*f*_ = 10.0 ± 0.1 cm) of ^90^Y^3+^ recovered in the eluate. Ultimately, the sorbent packed column also presented high stability for reusing 2-3 cycles without drop in its efficiency (±5%) towards Y^3+^ uptake and relative chemical recovery. A proposed flow sheet describing the analytical procedures for the separation of ^90^Y^3+^ from ^90^Sr^2+^ using chelating Chelex 100 (anion exchange) packed column is also included.

## 1. Introduction

In nuclear medicine, ^90^Y is an important radioisotope due to its satisfactory physical characteristics that include *β*^−^ emissions that allow tissue diffusion to a moderately extensive range, a suitable half-life (*t*_1/2_ = 64.4 h) and a nonradioactive daughter [1–6]. The United States-food and drug administration (US-FDA) has accepted Zevalin drug that incorporates ^90^Y [7, 8]. So, it is essential to the ^90^Sr impurity in ^90^Y samples owing to the radiotoxicity of ^90^Sr [9, 10]. ^90^Y decays to the stable ^90^Zr daughter, thus it is used as a pure *β*-emitter. High complex formation constants of Y^3+^ with complexing ligands make ^90^Y^3+^ valued in preparing radiopharmaceutical reagents [11, 12]. In light of the perceived need to abolish the radiotoxicity risk poses by ^90^ Sr, accurate separation, accurate, and reliable removal of ^90^Sr impurities from Y samples is of great importance. Therefore, great attention has been oriented towards establishing selective and low cost methods for chromatographic separation of ^90^Sr^2+^ from ^90^Y^3+^ samples with good radiochemical and radionuclide purity.

Recently, huge volumes of radioactive wastes and a diversity of radionuclides are generated from nuclear reaction process are certainly released into the natural environment [13, 14]. Thus, many approaches have been described in the last two decades for complete separation of ^90^Y^3+^ from various solutions via 2-ethylhexyl phosphonic acid mono-2-ethylhexyl ester (PC88A) in kerosene, [15], solvent extraction [16–20], organic resin and chelating agent and TBP-treated resins such as amberlite XAD-4 [21–24], cation ion exchanger [25, 26], pyridyl azo napthol (PAN)/zeolite composite [27], Teflon grain support di (2-ethyl hexyl) phosphoric acid-dodecane [28], Chelex-100 [29], paper chromatography [30], nafion-117, and dowex 50W X8 [31]. A ^90^Y generator system contained two extraction columns and sec-octylphenoxy acetic acid and tri-n-butyl phosphate as modifier have been used for separation of ^90^Y from ^90^Sr [32–34]. ^90^Sr/^90^Y generator elutes ^90^Y free from ^90^Sr with relative chemical recovery over 90% [35, 36]. Inorganic ion exchangers, e.g., cerium (IV) iodotungstate [37], zirconium-vanadte gel [38, 39] and chelating Chelex-100 ion exchange [10] in several steps have been used for complete separation of ^90^Y^3+^ from ^90^Sr^2+^ [31–34]. However, some of these methods have many drawbacks and limitations such as complexity, high cost, the necessity for suitable operation, and preconcentration due to their low capacity. Therefore, searching for establishing low cost, simple systems with short analytical time and ruggedness for chromatographic separation of Y^3+^ from Sr^2+^ species in aqueous solutions with good radiochemical and radionuclide purity are much sought after [37, 38].

With this background in mind, taking into account the importance of highly pure ^90^Y^3+^, the characteristics of the chelating group iminodiacetate moieties of the Chelex-100 anion ion exchanger and some of the synthesized inorganic sorbents as ideal and ecofriendly solid sorbents and in continuation to our previous study [10, 38–43], the current study is aimed to (i) revising the sorption profiles of Y^3+^ and Sr^2+^ onto the anion ion exchanger as a new candidate solid sorbent, (ii) establishing a simple and low cost and selective chromatographic separation of ^90^Y from its parent ^90^Sr by chelating Chelex-100 (anion ion exchanger) sorbent packed column and finally, and (iii) testing the reusability of the established chelating Chelex-100 sorbent packed column towards separation of Y^3+^ from its parent Sr^2+^ species in aqueous solutions. These results are supportive to recognize the analytical utility of the Chelex-100 in nuclear medicine, waste management and to properly assign the physicochemical behavior of radionuclide ^90^Y^3+^ and ^90^Sr^2+^.

## 2. Materials and Methods

### 2.1. Chemicals

Analytical reagents chemicals were used as received. High-density polyethylene (HDPE) bottles and all glassware's were immersed in hot detergent for 24 h and soaked in the acid mixture of HCl (50% v/v)-conc. HNO_3_ (3.0 M) at 1 : 1 v/v ratio, washed with double distilled water and finally dried in oven at 80–90°C. Stock solutions (1.0 M) of Sigma-Aldrich diethylenetriaminepenta acetic acid (DTPA), ethylenediaminetetra acetic acid (EDTA), HCl, HNO_3_, acetic acid, citric acid, and NaCl were prepared in deionized water. The chelating Chelex-100 (anion exchanger) (100–200 mesh) was purchased from BDH (Poole, England) and was used after washing 3-4 times with deionized water before use. ^89^Sr tracer was used as a substitute for ^90^Sr and the radio tracers ^90^Y^3+^ and ^90^Sr^2+^ were acquired by exposing the target Y_2_O_3_ and SrCO_3_ (99.9% purity) in Al container at an average thermal neutron flux density of 1.3 × 10^13^ neutrons/cm^2^/s at the ERR-1 research reactor (Atomic Energy Authority, Inshas, Egypt). Beta counting (liquid scintillation) was used to check ^90^Sr^2+^ efficiency. Millipore water (resistivity 18.2 MΩcm) was used in all experiments. The test solutions were prepared by spiking 100 mL of the water sample with a certain amount of YCl_3_ (1.771 × 10^−2^ M) and SrCl_2_ (1.323 × 10^−2^ M) individually.

### 2.2. Apparatus

A Geiger–Muller (*β*^−^ Counter) and window detector and a Scaler Ratementer SR7 (*γ*-Scintillation Counter) were used for ordinary gamma ray counter and it is fixed with well NaI (Tl) crystal. A high purity germanium (HPGe) coupled to a calibrated multichannel gamma analyzer (Silena, Milan, Italy) was employed to test impurities in the irradiated Y_2_O_3_ and SrCO_3_ as reported earlier [10]. The activity of ^90^Sr activity was monitored as reported [10]. Standard radionuclides were prepared from a mixed source of the radioisotopes ^155^Eu (86.5 and 105.3 keV), ^57^Co (122.1 and 136.5 keV), and ^137^Cs (661.6 keV). A mechanical shaker (Corporation Precision Scientific, Chicago, USA) with a shaking rate of 10–250 rpm was used for performing batch experiments. A centrifuge Chermle Z 230 A of 5500 rpm speed was also used. A close-fitting glass Jar chromatography (40 cm L and 5 cm id) of Whatmann paper No 1 (3 cm × 30 cm), Milli-Q Plus water system (Millipore, Bedford, MA, USA), and glass columns of 10.0 cm length (8, 15, and 20.0 mm internal diameter) were used in flow experiments. A digital micropipette (5.0–100 *µ*L) and a Jenway pH meter (model 3510) were used for the preparation and measuring the pH of more diluted working solutions, respectively.

### 2.3. Preparation of the Radioactive ^90^Y and ^09^Sr Tracers

An accurate mass of SrCO_3_ (200 mg, 99.9% purity, MW = 147.63) or Y_2_O_3_ (200 mg, 99.9% purity, MW = 225.8) was enfolded in pure Al foil precleaned with acetone and air dried before use to reserve the solid from possible contamination during cooling in the reactor. Al foil was then presented into another outer leak proof of Al and the target compound SrCO_3_ and Y_2_O_3_ were exposed for two days in the perpendicular channel of 2 MW water-cooled research reactor ERR-1 at average thermal neutron flux density of 1.3 × 10^13^ n/cm^2^s (Atomic Energy, Inshas, Egypt). The exposed target was left-hand to cool for 24 h before use. Accurate masses of each exposed SrCO_3_ (200 mg) and Y_2_O_3_ (200 mg) were dissolved in HCl (50.0 mL, 2.0 M). The test solution of ^89^SrCl_2_ and ^90^YCl_3_ was heated to dryness and redissolved in deionized water (100 mL) to achieve the final concentrations of ^89^SrCl_2_ (1.323 × 10^−2^ M) and ^90^YCl_3_ (1.771 × 10^−2^ M), respectively. In the irradiated product, the impurities were then checked as reported earlier [10]. The specific activity (*S*) was then computed as reported earlier [40–42]. The purity of irradiated ^90^YCl_3_ was also confirmed from the decay shape over 3 half-lives (*t*_1/2_) period at neutron flux density of 1.3 × 10^13^ neutrons/cm^2^/s as reported [44, 45].

### 2.4. General Batch Extraction Procedures

In a series of precleaned penicillin bottle, accurate masses (0.100 ± 0.002 g) of the precleaned Chelex-100 (Anion ion exchanger) were transferred and equilibrated with 20.0 mL solutions containing known concentrations (1.0 × 10^−5^ M) of YCl_3_ or SrCl_2_ in acetic acid, DTPA, EDTA, citric acid, or NaCl (1.0 × 10^−3^ M). The test solutions were then shaken for 60 min at various pH at 25°C. The solid phase extractor in each solution was allowed to settle down and an accurate volume (1.0 mL) of the aqueous phase of each solution was separated out. The radioactivity of ^90^Y and ^90^Sr, the relative extraction percentage (%*E*) and the amount (*q*_*e*_) of Y^3+^ and/or Sr^2+^ between the sorbent phase and the aqueous solution were then computed from their activities before and after extraction as reported [38, 39]. The distribution ratio (D, mL/g) of Y^3+^ and/or Sr^2+^ were also calculated using the following equation [43]:

(1) $$\begin{array}{c} K_{d}=\left(\frac{\%E}{100-\%E}\right)\times \frac{V\left(mL\right)}{W\left(g\right)}\text{ mL}/g, \end{array}$$

where *V* is the volume of solution (mL) and W is the mass of the dry ion exchanger (g). The quantity (*q*_*e*_) of Y^3+^ extracted per unit mass of the sorbent (mol g^−1^) was then calculated as reported earlier [39].

### 2.5. Separation of Y^3+^ from Sr^2+^ by Chelex-100 (Anion Exchanger) Packed Column

An accurate mass (1.0 ± 0.002 g) of the Chelex-100 (anion exchanger) sorbent was homogeneously packed in glass column (10.0 cm length × 0.8 cm i.d). An aqueous solution of acetic acid (1.0 × 10^−3^ M) of pH 1-2 was introduced into the sorbent packed column and quartz wool was then placed at the top of the resin after the sorbent had established down. This step helps in avoiding the disturbance of the resin particles during percolation of the test solution. Column was then washed with water 2-3 times at a 2.0 mL/min flow rate. The test solution (25 mL) containing Y^3+^ and Sr^2+^ and DTPA (1.0 × 10^−3^ M) was permeated to pass through the column at a 2.0 mL min^−1^ flow rate. Y^3+^ was only sorbed quantitatively, whereas Sr^2+^ species were passed through the column without sorption as specified from the radioactivity measurement of ^90^Y^3+^ and ^90^Sr^2+^ in the effluent. The sorbed Y^3+^ species were then recovered from the sorbent packed column with HNO_3_ (10 mL, 1.0 × 10^−1^M) at a 2.0 mL min^−1^ flow rate. The recovered Y^3+^ solutions were heated to dryness, redissolved in ultra-pure water, and the Y^3+^ purity was finally determined via computation of the half life (*t*_1/2_) as reported [39, 44]. Moreover, the influence of other parameter such as flow rates (1.0–5 mL min^−1^) and the internal diameter (0.8, 1.5, and 2.0 cm) on the analytical performance of Chelex-100 packed column for separation of ^90^Y^3+^ from ^90^Sr^2+^ was also examined.

### 2.6. Determination of Radiochemical and Radionuclidic Purity of ^90^Y

^90^Y purity was critically checked as follows: On a strip of Whatmann No. 1 paper, a drop of 5.0 *µ*L was put on the lower end of the chromatographic paper. After the spot has dried, the strip was immersed at its lower end in TRIS buffer (0.1 M) of pH 7 as a developer using ascending chromatograph technique without reaching the spot. The paper was left for 5-6 min to develop; the solution was then reserved out and the paper was allowed to dry. The paper was divided into equal parts (1.0 cm sections) and GM was used for counting *β* activity and the sorption factor (*R*_*f*_) was then computed. The radionuclidic purity of ^90^Y^3+^ in the eluate was determined from the purification factor (*P*_*f*_) = *A*/*A*_*o*_, where *A* and *A*_*o*_ are the activity of ^89^Sr in the eluate and solution, respectively. The radionuclidic purity was also computed from the decay curve over a period of at least 3 half-lives. The decay curve of ^90^Y^3+^ was planned by detecting *β*^−^ activity at one day intervals for 10 days after elution [46, 47].

## 3. Results and Discussion

### 3.1. Preliminary Study on the Sorption Profile of Y^3+^ and ^89^Sr^2+^ onto Chelex-100

The majority of ion exchangers using organic resin and chelator/complexing agent are simple and fast for separation of elements. However, they do not offer a ready-to-use eluate [3, 5]. Introductory study on Y^3+^ and Sr^2+^ uptake from the aqueous solution by chelating Chelex-100 (anionic form) displayed significant Y^3+^ sorption in a short time. Thus, a detailed study on the sorption profile of Y^3+^ and Sr^2+^ from the aqueous solution onto the established Chelex-100 sorbent was critically studied.

### 3.2. Programming of the Analytical Parameters

#### 3.2.1. Impact of pH of the Extraction Media

**Figure 1:**
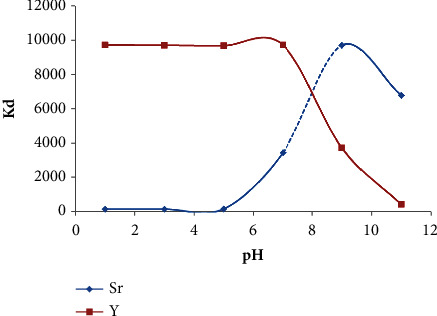
Plot of *K*_*d*_*versus* pH of Y^3+^ and Sr^2+^ sorption onto Chelex-100 (anion ion exchanger) from aqueous acetic acid solution (1.0 × 10^−3^ M) after 60 min shaking time at 25 ± 0.1°C.

**Figure 2:**
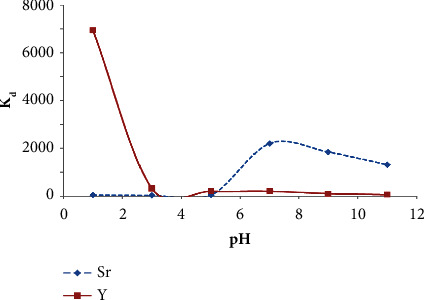
Plot of *K*_*d*_*versus* of pH of Y^3+^ and Sr^2+^ sorption onto Chelex-100 (anion ion exchanger) from aqueous DTPA solution (1.0 × 10^−3^ M) after 60 min shaking time at 25 ± 0.1°C.

The pH has a significant influence on the retention capacity for the ion exchange materials since the electrostatic interactions are the driving forces. Thus, in batch experiment, the uptake of Y^3+^ and Sr^2+^ (1.0 × 10^−5^ M) as YCl_3_ or SrCl_2_ from the test aqueous solutions containing DTPA, EDTA, acetic acid, citric acid, or NaCl (1.0 × 10^−3^ M) by Chelex-100 sorbent was studied over a wide range of solution pH (pH 1–11) after a shaking period of 60 min. In the aqueous phase, the amount of Y^3+^and Sr^2+^ was then determined. The sorption data of Y^3+^ and Sr^2+^ from the various extraction media into Chelex-100 (anionic form) are summarized in Table 1. In acetic acid and DTPA media, the sorption profiles of Y^3+^ and Sr^2+^ are also illustrated in Figures 1 and 2, respectively. In acetic acid media, the K_d_ of Y^3+^ sorption onto Chelex-100 sorbent reached a maximum value at pH 1–6 (*K*_*d*_ = 9930.6 ± 12.4), whereas the *K*_*d*_ gradually decreased on increasing the solution pH and reached minimum value (*K*_*d*_ close to zero negligible value) at pH11 as shown in Figure 1. On the other hand, Sr^2+^ uptake was insignificant in the pH range pH 1–5 (*K*_*d*_ = 0.0) and it gradually increased on growing the pH and reached maximum value at pH 9 (*K*_*d*_ = 9910.6 ± 10.6) and levelled off at higher pH up to pH 11 (*K*_*d*_ = 6000.7 ± 9.3) as shown in Figure 1. The sorption selectivity of Y^3+^ at pH 1–3 onto chelating Chelex-100 sorbent in the various extraction media followed the order: NaCl (*K*_*d*_ = 12278.5 ± 13.8 > acetic acid (*K*_*d*_ = 9930.6 ± 12.4) > EDTA (*K*_*d*_ = 9905.3 ± 5.4) > citric acid (*K*_*d*_ = 9886.4 ± 5.8) > DTPA (*K*_*d*_ = 7004.7 ± 3.3). At pH 1–3, Sr^2+^ species did not retained except in NaCl, where *K*_*d*_ = 66654.6 ± 5.7 (Table 1).

**Scheme 1:**
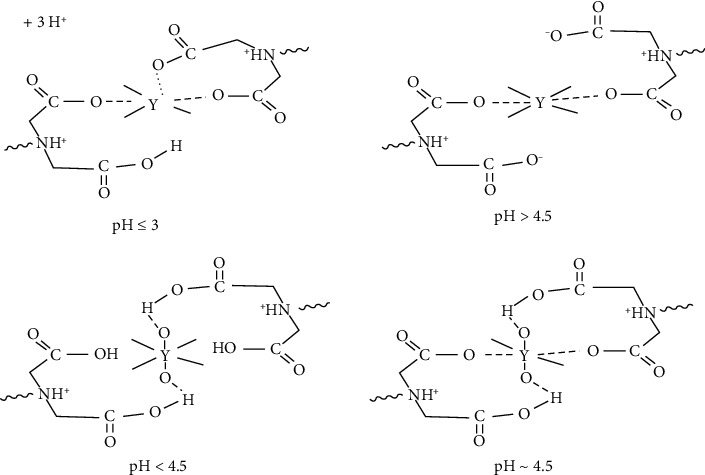
A scheme describing the possible interactions between Y^3+^ ions and the iminodiacetate moieties of Chelex -100 at various solution pH forming different complex species of Y^3+^ with Chelex-100 (anion ion exchanger).

In DTPA or acetic acid media of pH ranging from pH 1 to pH 4, Y^3+^ species were retained quantitatively onto the chelating Chelex-100 ion exchanger sorbent and the values of *K*_*d*_ were reproducible compared to EDTA, citric acid, or NaCl. Representative plot of *K*_*d*_*versus* of pH of Y^3+^ and Sr^2+^ sorption onto Chelex-100 (anion ion exchanger) from aqueous DTPA solution (1.0 × 10^−3^ M) after 60 min shaking time at 25 ± 0.1°C is shown in Figure 2. The observed behavior in Figure 2 is most likely attributed to the possible formation of nonpolar complex species of Y^3+^ species (YCl_6_)^3−^ with the available iminodiacetate moieties of the chelating Chelex 100 sorbent at pH ≤ 3 (pK_a1_ = 3.2) [40, 42, 43]. At low pH (pH ≤ 3), the possible interaction between the formed complex anion of yttrium (YCl_6_)^3−^ and the protonated iminodiacteate moieties of the chelating Chelex−100 anion ion exchanger by forming ternary complex ion associate may also contributed in the observed trend at low pH ≤ 3 [42, 43]. On the other hand, at pH above pH 3, one of the two carboxylic acids of the iminodiacetate moiety is deprotonated carrying a negative charge which attracts other positive cation present in extraction media, e.g., Na^+^ (introduced from pH adjustment by diluted NaOH which compete effectively with Y^3+^ because of their considerably higher concentration in solution [42]. The fact that, in acidic solutions, the N atom of the iminodiactic group retaining free electron pair is protonated, hence the resin is most likely can acts as weakly basic anion exchanger [43]. In addition, deprotonation of the second carboxylic group of the iminodiacetic moieties could also be proceeded at pH > 7, resulting in destabilization of the “guest-host” complexes between Y^3+^ and aminocarboxylic moieties [43]. This exchanger is also commonly regarded as an amphoteric ion exchanger and its ion exchange function depends on the solution pH that in contact with the resin as presented in Scheme 1.

In DTPA, EDTA, acetic acid, citric acid, or NaCl medium at pH > 7, Chelex-100 sorbent displayed good retention towards Sr^2+^ and the extraction profile of Sr^2+^ followed the order: NaCl (*K*_*d*_ = 12276.9 ± 5.7> citric acid (*K*_*d*_ = 9759.6 ± 5.7) > acetic acid (*K*_*d*_ = 7212.2 ± 5.8) > EDTA (*K*_*d*_ = 3623.4 ± 3.7) > DTPA (*K*_*d*_ = 1614.5 ± 5.2) was achieved. On the other hand, at pH > 7, the chelating Chelex-100 sorbent displayed no affinity towards Y^3+^ from citric acid, EDTA or DTPA. The fact that the chelating agents EDTA and DTPA act as competitors having similar groups with the Chelex-100 sorbent and both are able to form complexes in solution with Y^3+^ and Sr^2+^ preventing their adsorption while acetic acid is the weakest medium [42, 48]. This behavior is most likely attributed to the strong and weak ion-association interaction of the accessible specific active sites of the Chelex-100 solid extractor towards Sr^2+^ and Y^3+^, respectively, as reported the authors in [42, 48]. On the other hand, it may be thought that for smaller molecules a more pronounced difference between the adsorption sites on the surface of Chelex-100 and inside the sorbent pores as reported [48]. In acetic acid, EDTA or NaCl at pH > 7, separation of Y^3+^ from Sr^2+^ was not complete. Thus, in Y^3+^ separation from Sr^2+^, acetic acid, or DTPA (1.0 × 10^−3^ M) was implemented as a preferred extraction medium at lower pH in the subsequent study.

#### 3.2.2. Impact of Shaking Time

The influence of shaking time over 0.0–2.0 h on Y^3+^ uptake from aqueous acetic acid or DTPA solution (1.0 × 10^−3^ M) onto Chelex-100 was studied. Y^3+^ sorption onto the ion exchanger was fast at the initial stage and attained maximum sorption percentage after 60 min shaking time and remained constant at extra time. This trend was supported from the value of half life (*t*_1/2_) of Y^3+^ retention via the plots of log *C*_*t*_/*C*_0_ of Y^3+^ versus shaking time. The value of the half-life (*t*_1/2_) of Y^3+^ retention from the aqueous acetic acid or DTPA solution as computed from the plot of log *C*_*t*_/*C*_0_ of Y^3+^ versus shaking time was in the range 2.42 ± 0.05 min. Representative plot is given in the Supplementary Description (SD.  1). Thus, the rate-controlling step for Y^3+^ sorption by the sorbent is not only gel diffusion control as in the ion exchangers [49, 50]. At the initial stage of shaking time, the plot of %*E* of Y^3+^ versus log time was fast and linear approving the occurrence of intraparticle diffusion [10, 50]. Thus, a 60 min shaking time was adopted in the following study.

#### 3.2.3. Influence of Media Polarity

**Figure 3:**
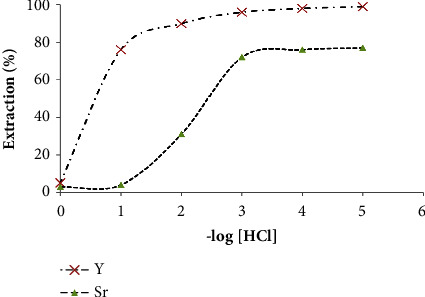
Plot of the extraction percentage (%) of Y^3+^ and Sr^2+^ (1.0 × 10^−5^ M) as YCl_3_ or SrCl_2_ onto Chelex-100 (anion ion exchanger) versus–log([HCl) concentrations (1.0 × 10^−5^–1.0 M) in the aqueous media after 60 min shaking time at 25 ± 0.1°C.

The extraction medium in solid phase extraction procedures has a pronounced effect on the performance of Y^3+^ separation. Thus, Y^3+^ and Sr^2+^ ions uptake from the test aqueous solutions (20.0 mL) containing various known concentrations (1 × 10^−5^–1.0 M) of HCl or HNO_3_ at standard concentration of Y^3+^ and Sr^2+^ (1.0 × 10^−5^ M) was studied over a shaking time of 60 min at room temperature. At equilibrium, the remained Y^3+^ and Sr^2+^ ions in the aqueous phase was measured and the extraction percentage (*E*, %) and the *D* were then computed as reported [10]. In HCl media, the data are presented in Figure 3, where the *E*% and *D* of Y^3+^ and Sr^2+^ by the sorbent decreased on rising HCl concentration from 1.0 × 10^−5^ to 1.0 M. At HCl concentration ≥1.0 × 10^−1^ M, Sr^2+^ species were not retained, while 75.0 ± 2.1% of Y^3+^ was retained. The strong interaction of the active sites of the sorbent with Y^3+^ may account for this trend [51, 52]. The strong binding of Y^3+^ to form [YCl_6_]^3−^ complex species [53] compared to Sr^2+^ in HCl media may also account for the observed trend. In HNO_3_ (1.0 × 10^−5^–1.0 M), Y^3+^ species did not sorbed onto chelating Chelex-100 sorbent whereas significant sorption of Sr^2+^ (*K*_*d*_ = 650.4 ± 3.64 mL g^−1^) was noticed at 1.0 × 10^−5^ M and decreased on rising HNO_3_ concentration up to 1.0 M (*K*_*d*_ = 210.2 ± 3.64 mL g^−1^). The average chemical extraction percentage of ^89^Sr and ^90^Y from acetic acid (1.0 × 10^−3^ M) at different solution pH onto Chelex-100 was also studied. The results are illustrated in Figure 1 where in acetic acid media at pH ≤ 5, Y^3+^ species were retained quantitatively while Sr^2+^ ions did not get sorbed. However, in the subsequent study, HNO_3_ (1.0 × 10^−1^ M) was nominated as a prober reagent for Y^3+^recovery from Chelex-100 sorbent packed column since it is easily evaporated by gentle heating.

### 3.3. Possible sorption Mechanism for Y^3+^ Retention

The affinity of the sorbent towards Y^3+^ played an important role on its uptake. The nature and number of the specific sorbent sites are involved instantaneously in Y^3+^ uptake from the solution [16]. The chelating Chelex-100 sorbent acts as an active “weak anion-exchanger” towards complex species of Y^3+^ such as (YCl_6_)^3−^ in HCl media [53] and “liquid-liquid extraction” with the salt performing as salting-out reagent in Y^3+^ uptake. The salt added decreases the water molecules available to solvate Y^3+^ ions which would be required out of the solvent onto the sorbent phase. Thus, water structure enforced ion pairing is somewhat the driving force for Y^3+^ uptake and “surface adsorption” effectively take part in the Y^3+^ extraction [53, 54]. Based on the obtainable results and the data reported earlier [54, 55], a dual sorption mechanism involving absorption related to “weak-base anion exchange” and “solvent extraction” in addition to “surface adsorption” of Y^3+^ is proposed. Thus, retention mechanism of Y^3+^ can be stated by the following equation [54, 55]:

(2) $$\begin{array}{c} C_{r}=C_{abs}+C_{ads}=DC_{aq}+\frac{SK_{L}C_{aq}}{1}+K_{L}C_{aq}, \end{array}$$

where *C*_*r*_ and *C*_*aq*_ are the equilibrium concentrations of Y^3+^ ions onto the sorbent and in solution, respectively. *C*_*abs*_ and *C*_*ads*_ are the equilibrium concentrations of Y^3+^ absorbed and adsorbed onto the sorbent while *S* and *K*_*L*_ are the parameters of the Langmuir adsorption model [54, 55].

### 3.4. Separation of ^90^Y^3+^from Sr^2+^ by Sorbent Chelex-100 (Anion Form) Packed Column

An aqueous solution (25.0 mL) composed of acetic acid (1.0 × 10^−3^ M), Y^3+^ and Sr^2+^ ions (1.0 × 10^−5^ M) was permeated through Chelex-100(Anion exchanger) packed column at a reasonable flow rate (2.0 mL min^−1^). Y^3+^ species were retained quantitatively whereas Sr^2+^ ions were passed without uptake as revealed from ICP-OES determination of ^90^Y^3+^ and ^90^Sr^2+^ ions in the effluent *versus* reagent blank. Selection of proper eluting agent prior to use of ^90^Y^3+^ for labeling and radiolysis of organic support materials is crucial and is identified as the main limitations of current 90Sr/90Y [35, 56]. Thus, the established methodology offered a facile, better selectivity and simple approach compared to the published work [12–21, 57]. Numerous eluting agents such as HNO_3_, HClO_4_, H_2_SO_4_, and acetic acid (1.0 × 10^−1^M) were checked for recovery of Y^3+^ from chelated Chelex-1000 packed column. Among these reagents, good percentage recovery (99.5 ± 2.9%) of ^90^Y^3+^ was only achieved with HNO_3_ (10 mL, 1.0 × 10^−1^ M) as a prober agent for Y^3+^ recovery at a 1.0 mL/min flow rate using Chelex-100 sorbent packed glass column of 8 mm internal diameter. On the other hand, HNO_3_ can easily remove from the recovered ^90^Y^3+^ solution by gentle evaporation. The solid residue was redissolved in deionized water and analyzed as reported [56].

Moreover, the impact of the internal column diameter (0.8, 1.5, and 2.0 cm) on the performance of chelated Chelex-100 packed column on the separation of Y^3+^ from Sr^2+^ ions was examined at a 1.0 mL min^−1^ flow rate. Acceptable separation and relative chemical recovery of Y^3+^ from Sr^2+^ was only achieved at 8 mm internal diameter of the column, whereas at internal diameter greater than 8 mm, Y^3+^ recovery was not complete (<90%) The influence of the flow rate (1.0–5 mL min^−1^) on Y^3+^ separation from ^9^Sr^2+^ ions was critically tested. Good separation with acceptable relative chemical recovery (over 99%) of Y^3+^ from Sr^2+^ ions was achieved at a flow rate of 1.0 mL min^−1^. Thus, in the subsequent study, the flow rate and the internal diameter of the Chelex −100 sorbent packed column were adopted at a 1.0 mL min^−1^ flow rate and 8 mm internal diameter.

### 3.5. Radiochemical Purity of ^90^Y^3+^

**Figure 4:**
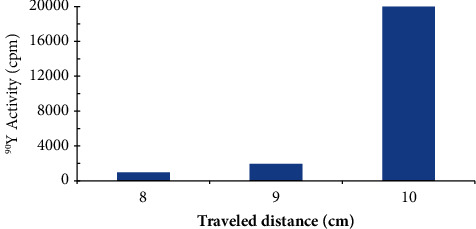
Plot of radioactivity of ^90^Y^3+^ species in the eluate *versus* travelled distance.

Validation of Chelex-100 (anion form)-packed column for chromatographic separation of ^90^Y from ^90^Sr was critically tested by calculating the retardation factor (*R*_*f*_) from the radio chromatogram of ^90^Y on the original spot constructed by plotting radioactivity (cpm) versus travelled distance, cm. The data are presented in Figure 4 and the *R*_*f*_ value was 10.0 ± 0.1 cm of total activity on the original spot in agreement with the data published earlier [31–36]. These data also signify that over 99.2 ± 2.1% of ^90^Y^3+^ species are present in the eluate as ^90^YCl_3_ as reported by the authors in [46, 56].

### 3.6. Radionuclides Purity of ^90^Y^3+^

**Figure 5:**
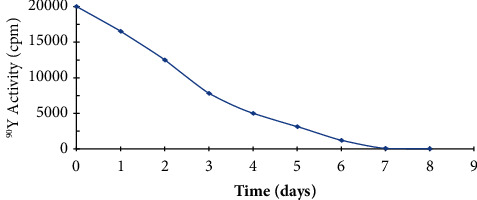
Plot of the radioactivity of ^90^Y^3+^ species *versus* time.

**Figure 6:**
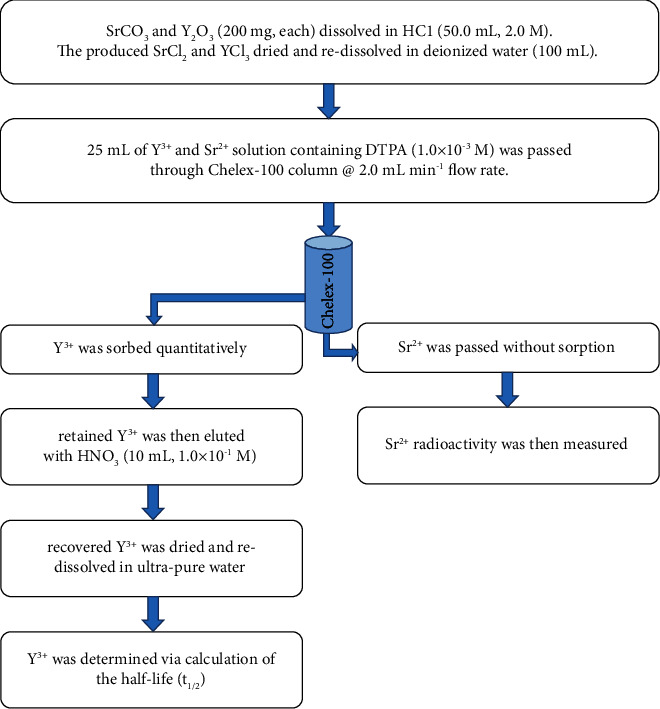
A proposed flow sheet of the analytical procedures for the separation of ^90^Y^3+^ from ^90^Sr^2+^ using Chelex 100 (anion exchange) packed column.

The proposed protocol was tested by measuring the radionuclidic purity using the purification factor (*P*_*f*_=*A*/*A*_*o*_), where *A* and *A*_*o*_ are the ^90^Sr^2+^ activity in the recovered and loaded solution, respectively. The *P*_*f*_ value was lower than 1.1 × 10^−6^, demonstrating negligible impurity of ^90^Sr^2+^ in ^90^Y^3+^ solution [45, 46]. The radionuclidic purity of ^90^Y^3+^ was also computed from radioactivity (cpm) plot of ^90^Y^3+^ in solution versus time (day) (Figure 5). The value of half life (t_1/2_) of ^90^Y as computed from the decay curve (Figure 5) was found equal 64.4 h in good agreement with the data reported earlier [38, 39], revealing high purity of ^90^Y^3+^ with good performance of Chelex-100-packed column towards ^90^Y^3+^ separation from ^90^Sr^2+^. The whole analytical procedures for ^90^Y^3+^ separation from ^90^Sr^2+^ by Chelex−100 sorbent is presented in the proposed flowsheet (Figure 6).

## 4. Conclusion, Drawbacks, and Future Outlooks

In summary, the current study presented an optimized protocol for selective separation of ^90^Y^3+^ from ^90^Sr^2+^ with good purity using chelating Chelex-100 (anion exchanger) packed column. The membrane-like structures and the available active sites of the Chelex-100 solid extractor permit good separation of Y^3+^ from Sr^2+^ compared to other sorbents [12–22, 57]. Compared to previous methods for separation of ^90^Y^3+^ from ^90^Sr, Chelex-100 requires slight sample operation to reduce the analysis time, and it does not require solvent evaporations and reconstruction step. This method displays high selectivity for separation of ^90^Y^3+^ from ^90^Sr^2+^ at the low level. The purity of ^90^Y can be tested by quality control procedures. The established extractor looks low cost and valuable alternative sorbent over the common rigid or granular solid extractors. A dual sorption mechanism of Y^3+^ comprising both “surface adsorption” and an added component of “ion exchanger and/or solvent extraction” is anticipated. In addition, the results revealed the possible use of Chelex-100 sorbent packed column for complete enrichment and recovery of Y^3+^ for 2-3 times without significant decrease in its performance. Work is ongoing for studying the impact of memory effect, various organic materials in water samples and online enrichment of ultratrace levels of Y^3+^ from great volume of water samples followed by subsequent determination. The study also shows that the established extractor can be used as cheap, efficient and ecofriendly solid sorbent for Y^3+^ separation from Sr^2+^, whereas other methodologies have high operational costs and sometimes yield undesirable by-products when linked to physical and chemical methods. The fact that the use of one factor at a time has many drawbacks and shortcomings and the cooperating results of numerous features might advance the signal and the utility of the proposed methodology. Therefore, design experiment for separation of Y^3+^ from Sr^2+^ is suggested in the forthcoming study. The developed strategy provides new sorbents for establishing a method for radiochemical separation.

## Figures and Tables

**Table 1:** Influence of the extraction media (acetic acid, citric acid, DTPA, EDTA and NaCl), and the solution pH on the distribution ratio (*K*_*d*_) of Y^3+^ and Sr^2+^ towards retention onto Chelex 100 (anion form)^†^.

pH	Compound									
	Acetic acid		Citric acid		DTPA		EDTA		NaCl	
	Y^3+^	Sr^2+^	Y^3+^	Sr^2+^	Y^3+^	Sr^2+^	Y^3+^	Sr^2+^	Y^3+^	Sr^2+^
pH 1–pH 3	9930 ± 12.4	0.0	9886 ± 5.8	0.0	7004 ± 3.3	0.0	9905 ± 5.4	0.0	12278 ± 13.8	66654 ± 5.7
pH 4-pH5	9927.7 ± 9.3	0.0	123.5 ± 4.7	0.0	20.4 ± 2.9	0.0	7.3 ± 0.21	4.3 ± 2.5	12273 ± 10.4	4734 ± 12
pH 7	9924 ± 11.3	3924.4 ± 11.3	125.8 ± 5.7	2012.6 ± 3.7	20.4 ± 2.9	2000 ± 2.2	0.0	4.3 ± 0.2	12275 ± 15.1	1154 ± 5.1
pH 9	4027 ± 7.5	9120 ± 12.4	0.0	9654 ± 5.7	20.4 ± 2.1	1734 ± 2.2	0.0	4123 ± 2.3	1027 ± 9.5	12276 ± 5.7
pH 11	100.7 ± 3.7	7212.2 ± 5.8	0.0	9759 ± 5.7	0.0	1614.5 ± 5.2	0.0	3623 ± 3.7	11452 ± 9.5	12276 ± 5.7

^†^The concentration of acetic acid, citric acid, DTPA, EDTA, or NaCl is equal to 1.0 × 10^−3^ M.

## Data Availability

The data used to support the findings of this study are included within the manuscript and supplementary materials and also from corresponding author upon request.

## Supplementary Materials

Supplementary Materials SD. 1: rate of Y^3+^uptake from aqueous DTPA (1.0 × 10^−3^ M) solution using Chelex-100 sorbent.
