# Supplementary material for: Sorption Characteristics and Chromatographic Separation of 90Y3+ from 90Sr2+ from Aqueous Media by Chelex-100 (Anion Ion Exchange) Packed Column
Source: Int J Anal Chem. 2024 May 13;2024:6232381. doi: 10.1155/2024/6232381 (PMC11105960; doi:10.1155/2024/6232381)
Supplement: Supplementary Materials — SD. 1: rate of Y3+uptake from aqueous DTPA (1.0 × 10^−3^ M) solution using Chelex-100 sorbent. [file 6232381.f1.doc]

**Supplementary Description (SD)**

**
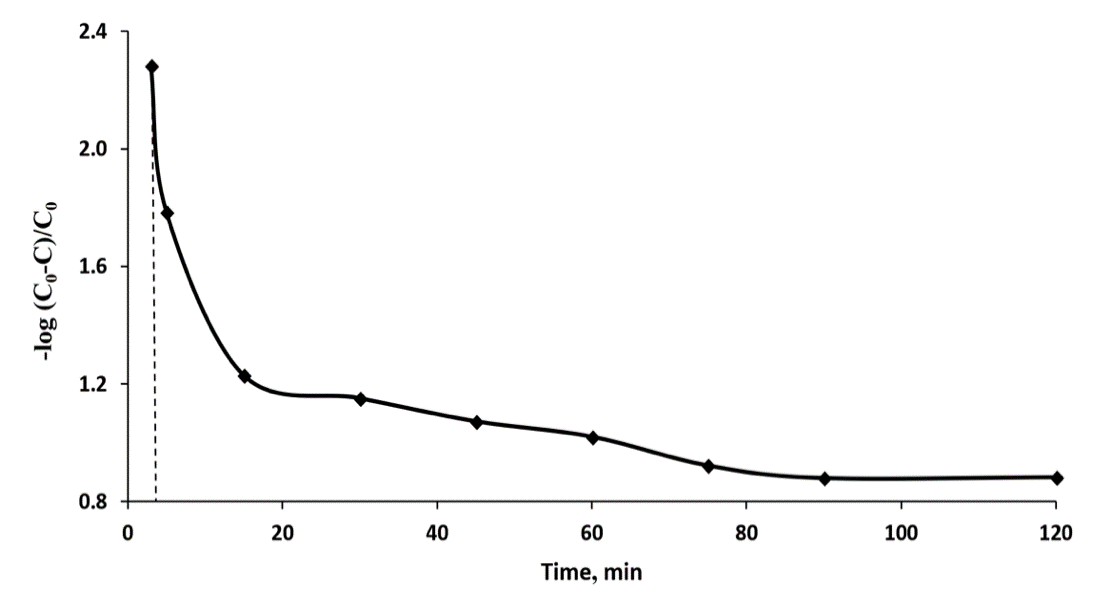
**

Supplementary description **(SD). SD. 1.** Rate of Y^3+^ retention from aqueous DTPA (1.0×10^-3^ M) solution into Chelex-100 sorbent. The computed value of the half-life (t_1/2_) of Y^3+^ retention from the aqueous DTPA from the plot of log C_t_/C_0_ of Y^3+^ *versus* shaking time is was in the range 2.42 ±0.05 min.
